# Cas9-guide RNA ribonucleoprotein-induced genome editing in the industrial green alga *Coccomyxa* sp. strain KJ

**DOI:** 10.1186/s13068-018-1327-1

**Published:** 2018-12-10

**Authors:** Yuya Yoshimitsu, Jun Abe, Shigeaki Harayama

**Affiliations:** 1 Advanced Research and Innovation Center, DENSO CORPORATION, Komenoki-cho, Nisshin-Shi, Aichi 470-0111 Japan; 2 Research and Development Initiative, Chuo University, Bunkyo-ku, Tokyo, 112-8551 Japan; 3 Department of Biological Sciences, Faculty of Science and Engineering, Chuo University, Bunkyo-ku, Tokyo, 112-8551 Japan

**Keywords:** Microalgae, *Coccomyxa*, Genome editing, Cas9-gRNA RNP, Electroporation

## Abstract

**Background:**

Oxygen-evolving photosynthetic microorganisms, collectively termed as microalgae, are gaining attention as alternative fuel sources. The unicellular alga *Coccomyxa* sp. strain KJ that belongs to the class Trebouxiophyceae can grow rapidly in minimal mineral media and accumulate triacylglycerols at levels > 60% (w/w) of its dry weight under nitrogen depletion conditions. Thus, the strain can be a good candidate for biofuel production. Still, substantial improvements in lipid productivity and other traits of this strain are needed to meet commercial production requirements. Consequently, the development of new genetic tools including genome editing that are applicable to this strain is highly desired.

**Results:**

In this paper, we report successful genome editing of strain KJ by intracellular delivery of a ribonucleoprotein complex comprising recombinant Cas9 protein and guide RNA. For introduction of Cas9-guide RNA ribonucleoprotein into strain KJ cells, we used an electroporator with a short (2.5 ms) electric pulse at a high field strength (7500 V cm^−1^) followed by multiple 50-ms electric pulses at low field strength (250 V cm^−1^). Under these conditions, we successfully isolated several knockout lines of the *FTSY* gene of strain KJ, encoding a signal recognition particle-docking protein at a frequency of 0.01%.

**Conclusions:**

Our study shows applicability of DNA-free genome editing in *Coccomyxa*, which may be applicable in other Trebouxiophyceae species.

**Electronic supplementary material:**

The online version of this article (10.1186/s13068-018-1327-1) contains supplementary material, which is available to authorized users.

## Background

Microalgae represent one of the most promising resources for biofuels because of their high efficiency in biomass production and their ability to grow on non-arable land. The class Trebouxiophyceae is one of the major groups in the division Chlorophyta and contains many species exhibiting high lipid contents. For example, *Botryococcus braunii* produces high levels of hydrocarbons and may be used as feedstock for biofuels [1–3]. *Chlorella vulgaris*, with a long history of use as a superfood supplement, accumulates lipids up to 53% of its dry weight under nutrient-depleted conditions [4]. *Parachlorella kessleri,* formerly called *C. kessleri*, and a cosmopolitan marine microalga *Picochlorum atomus* also accumulate lipids at > 50% of its dry weight [5, 6]. In particular, *Choricystis minor* cultivated under nitrogen and phosphorus starvation accumulates lipids at a level of almost 70% of its dry weight in 10 days [7]. Thus, the class Trebouxiophyceae comprises many interesting species that can be used for commercial production of biofuels. However, only limited resources for molecular breeding are available in this taxonomic group, in spite of *Chlorella* strains being considered to be ideal hosts for heterologous protein production [8].

The unicellular alga *Coccomyxa* sp. strain KJ (hereinafter referred to as “strain KJ”) that belongs to the class Trebouxiophyceae can rapidly grow in minimal mineral media and accumulate triacylglycerols in lipid bodies at levels > 60% (w/w) of its dry weight under nitrogen depletion conditions [9]. Strain KJ which has been isolated by Professor Hideaki Miyashita in the Rural Biomass Research Project granted from the Ministry of Agriculture, Forestry and Fisheries of Japan, can be cultivated in open ponds at pH between 3.0 and 4.0 under which the chance of contamination with other phototrophs and protozoa is minimized. Nonetheless, production costs of biofuels with this strain are estimated to be much higher than fossil fuels. For economic viability, costs need to be reduced by increasing lipid productivity of this alga through molecular breeding.

In our previous studies, we had developed basic and advanced genetic tools applicable to *Coccomyxa* spp., including methods for gene delivery, self-cloning, transgene removal using the *piggyBac* transposase system, and gene targeting with transcription activator-like effector nuclease [10–13]. Although these techniques provided insights into candidates for gene targeting to genetically improve *Coccomyxa* spp., clustered regularly interspaced short palindromic repeats (CRISPR)-mediated genome editing has still not been developed and may be a technique that is more effective than other genome editing techniques. Methods for delivering the Cas9 protein and guide RNA (gRNA) as a preassembled Cas9-gRNA ribonucleoprotein (RNP) complex (Cas9-gRNA RNP) have recently been developed [14–16]. These new methods to generate functional Cas9-gRNA complexes do not require the host cellular transcription/translation machinery unlike the original CRISPR methods where DNAs encoding the Cas9 protein and gRNA(s) are delivered. In addition, the Cas9-gRNA RNP method does not involve integration of foreign DNA sequences into the host genome; thus, knockout mutants obtained will be exempted from current genetically modified organisms legislation in many countries [17–19].

A DNA-based CRISPR/Cas9 technique was applied to the model green alga *Chlamydomonas reinhardtii*, but it failed to recover transformants containing the intact Cas9 gene [20]. From this observation, the authors suggested that functional expression of the Cas9 protein in *C. reinhardtii* is harmful to the host. Conversely, *C. reinhardtii* knockout derivatives could be isolated using Cas9-gRNA RNP [21–23], indicating that the delivery of Cas9-gRNA RNP overcomes cytotoxicity caused by stable Cas9 protein expression. We were then interested in applying the Cas9-gRNA RNP method to an industrial microalga, i.e., strain KJ. We report here on successful isolation of genome-edited strains of strain KJ after optimization of electroporation conditions.

## Results and discussion

### Optimization of genetic transformation of strain KJ with an ELEPO21 electroporator

After successful genome editing through Cas9-gRNA RNP in animal cells, we were interested in applying this technique in *Coccomyxa* spp. Although genetic transformants of *Coccomyxa* sp. strains Obi and KJ could be obtained through particle bombardment using Biolistic PDS-1000/HE (Bio-Rad) or through electroporation using Genepulser XCell™ (Bio-Rad) [10–13], the transformation efficiency was generally in the order of 10^−6^ transformants per input cell. Because electroporators developed by Nepa Gene achieve high transformation efficiency in *C. reinhardtii* cells with intact cell wall [24], we used the ELEPO21 electroporator for delivery of Cas9-gRNA RNP in strain KJ cells.

**Fig. 1:**
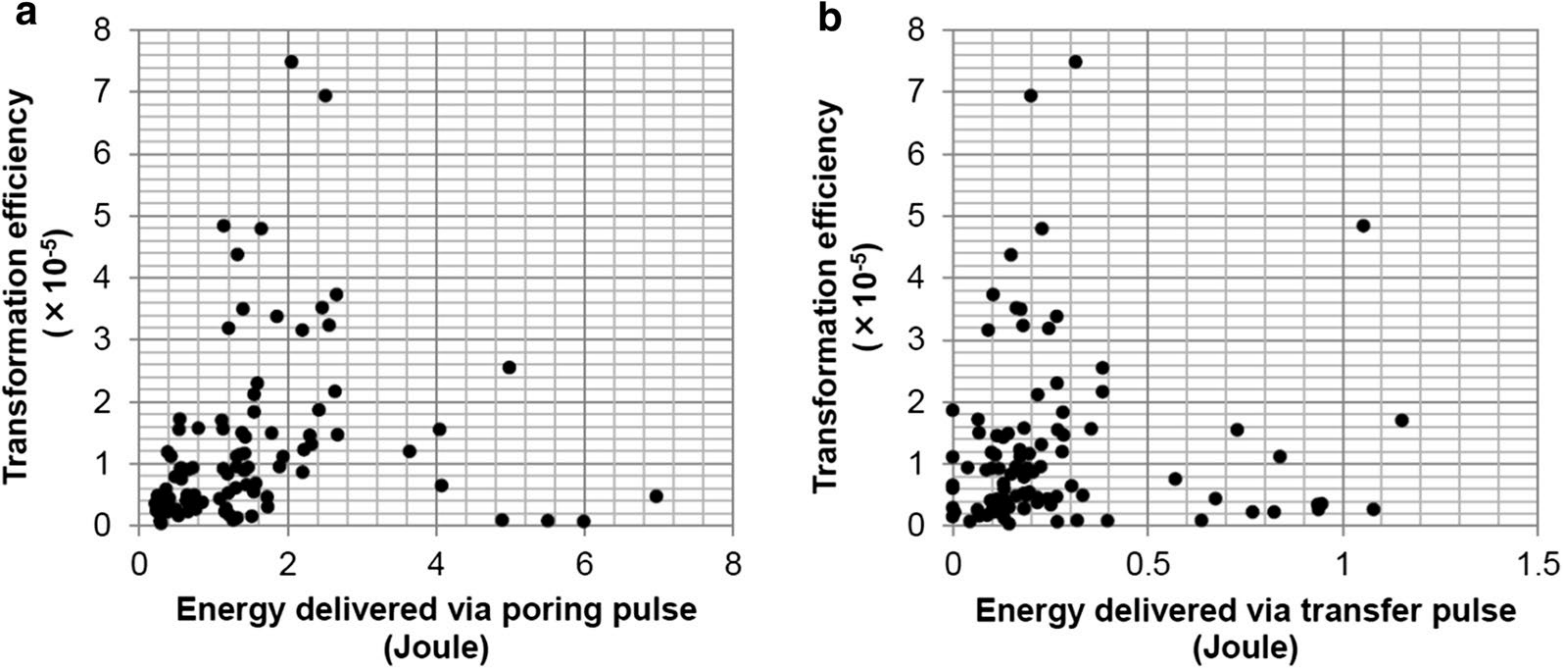
Effect of energy delivered via poring and transfer pulses on transformation efficiency of strain KJ. Energies delivered via poring pulse and transfer pulses were recorded for each electroporation. The number of Zeocin™-resistant clones obtained after electroporation of 3.0 × 10^7^ cells with 1, 2 or 4 µg DNA of the pble-PeEGFP-KE1E plasmid was counted in each experiment; the transformation efficiency was calculated by dividing the number of Zeocin™-resistant clones by the number of input cells (3.0 × 10^7^). A total of 97 electroporation experiments were performed; all 97 results (Additional file 1) are incorporated in each of the panels (**a**) and (**b**). **a** The relationship between the transformation efficiency and energy delivered via the poring pulse. **b** The relationship between the transformation efficiency and energy delivered via the transfer pulse

It is technically difficult to measure the efficiency of protein delivery by electroporation; therefore, we attempted to optimize electroporation conditions with the ELEPO21 electroporator by determining genetic transformation frequency of strain KJ, as described in the "Methods" section. Because the ELEPO21 apparatus can function with two types of electric pulses “poring” and “transfer,” we first aimed at optimizing the parameters for both pulses. Genetic transformation frequencies were determined when the following parameters were changed: for the poring pulse, we varied the electric field strength between 3000 and 11,500 V cm^−1^, the pulse width between 2.5 and 15 ms, and number of pulses between 1 and 4; while for the transfer pulse, we varied the electric field strength between 5 and 500 V cm^−1^ and the pulse width between 1 and 99.9 ms. A total of 97 electroporation reactions were performed in 29 experiments using cells from independent cultures. Analysis of the 97 datasets showed that the transformation efficiency using the same electroporation conditions was different when cells from different cultures were used, suggesting that cell competency was variable in different cultures; and thus comparisons were made between the data obtained with cells from the same culture to elucidate optimum electroporation conditions. We did not investigate growth conditions that optimize competence for DNA transformation in this study, but we are currently studying the relationship between cell-wall morphology and transformation efficiency as the cell wall is one of the barriers to exogenous DNA uptake, and its morphology changes during proliferation [25]. The electric field strength and duration of the poring and transfer pulses strongly influenced the transformation frequency (Fig. 1). Since electric energy delivered by pulses, but neither electric field strength per se nor pulse width per se, correlated with the extent of membrane permeabilization [26], electric energy in Joule delivered by each pulse was calculated by multiplying the measured values of current (ampere), voltage (volt per 0.2 cm), and pulse width in second during each discharge, and possible relationships between total electric energy delivered by poring or transfer pulses and transformation efficiency were examined by scatter plots. As shown in Fig. 1, increased electric energies tended to increase the transformation efficiency up to certain extent (2.4 Joules for poring pulse and 0.4 Joules for transfer pulse), and then tended to decrease (Fig. 1). This result suggested that if electric energy exceeded the optimal values, cell viability decreased due to cell membrane disintegration. This inference was confirmed experimentally: cell viability gradually decreased in an energy-dependent manner (Additional file 1: Figure S1). The highest genetic transformation efficiency, which was 7.5 × 10^−5^ transformants per input cell, was achieved under the following “optimum” conditions: poring pulse electric field strength of 7500 V cm^−1^, poring pulse width of 2.5 ms, poring pulse number of 1, transfer pulse electric field strength of 250 V cm^−1^, transfer pulse width of 50 ms, and harvesting cell density of 0.8 × 10^7^ cells mL^−1^. Consequently, transformation efficiency of *Coccomyxa* increased by ≥ 1 order of magnitude compared to that reported previously [10, 12].

### Delivery of fluorescently labeled Cas9 protein into strain KJ cells

We applied the established “optimum” electroporation conditions for delivery of fluorescently labeled Cas9 protein into strain KJ cells. However, arcing occurred at 7500 V cm^−1^ probably because of high salt concentration in the purified Cas9 protein solution. Therefore, we conducted desalination of the Cas9 protein solution, as described in the "Methods" section, and this approach was effective in preventing arcing.

**Fig. 2:**
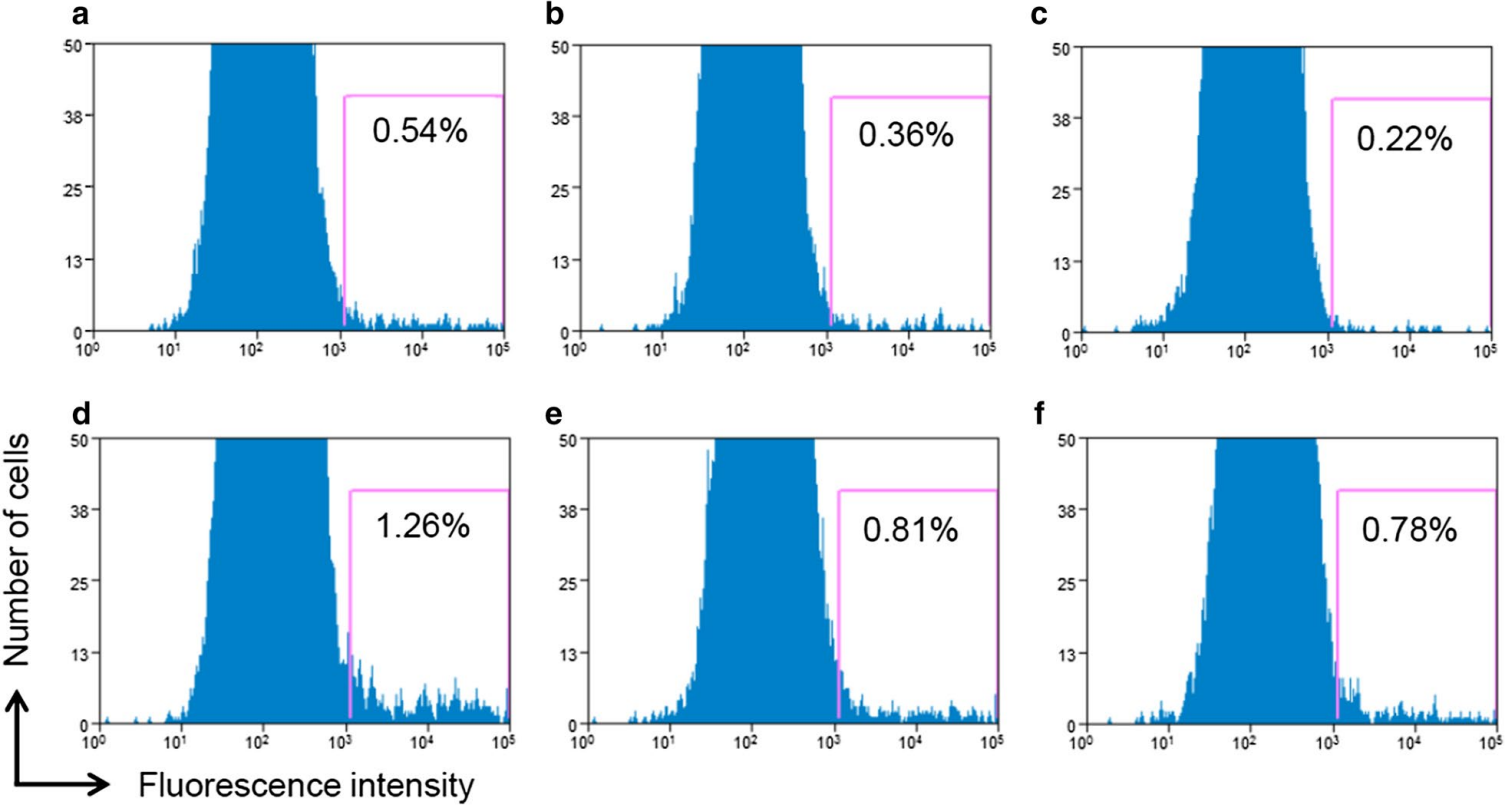
Detection of delivered fluorescently labeled Cas9 proteins within strain KJ cells. Fluorescently labeled Cas9 proteins were mixed with strain KJ cells in an electroporation cuvette; the cells were not electroporated (**a**, **b** and **c**) or electroporated using the “optimum” conditions described in the text (**d**, **e**, and **f**). Then, cells were analyzed by FACS without washing (**a** and **d**) or after three (**b** and **e**) or five washings (**c** and **f**) with PBS buffer to eliminate the fluorescently labeled Cas9 protein adsorbed on cell surface. The numbers within each panel are percentages of gated events (namely, detection of highly fluorescent cells)

To evaluate the efficiency of introduction of the Cas9 protein in strain KJ cells, HiLyte Fluor™ 555-labeled Cas9 protein was electroporated into the cells; the electroporated cells with or without washing with PBS buffer were analyzed by fluorescence-activated cell sorting (FACS). As shown in Fig. 2, majority cells emitted weak fluorescence (< 10^3^) of unidentified compound(s). The percentage of high fluorescence cells (> 10^3^) in both the electroporated and non-electroporated samples decreased with the number of cell washing with PBS buffer. After five washes, the population of cells with high fluorescence was 0.22% among non-electroporated cells, whereas it was 0.78% among electroporated cells (Fig. 2). These results implied that a small population (about 0.5%) of electroporated cells imported Cas9 protein inside the cell.

**Fig. 3:**
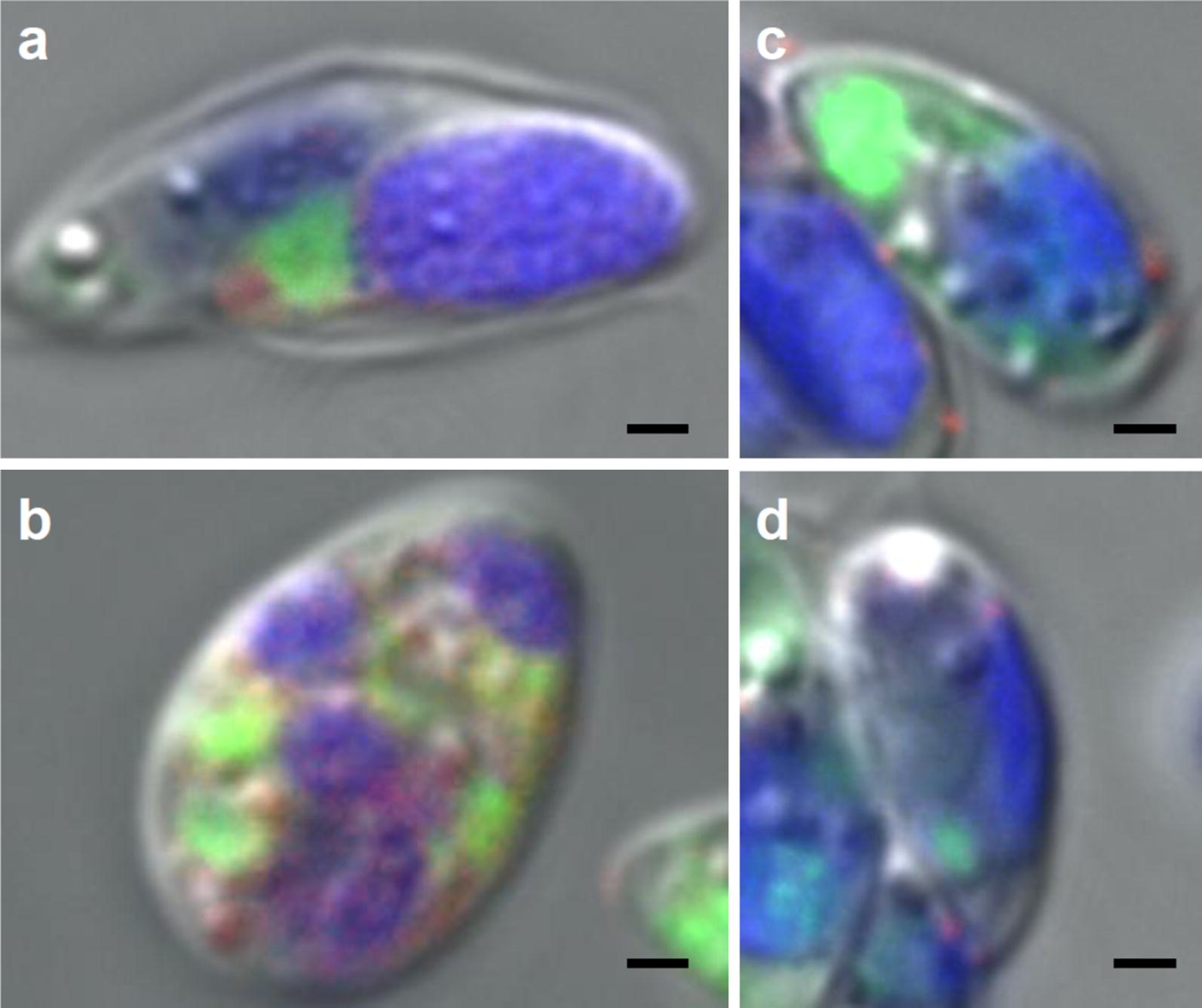
Localization of fluorescently labeled Cas9 proteins in strain KJ cells by confocal laser scanning microscopy. Fluorescently labeled Cas9 proteins were mixed with strain KJ cells in an electroporation cuvette; the cells were electroporated or not electroporated. Subsequently, the cells were washed five times with PBS buffer, and cells containing HiLyte Fluor™ 555-Cas9 were examined microscopically. Micrographs of two electroporated (**a**, **b**) and two non-electroporated (**c**, **d**) cells are shown. The micrographs represent pseudo-colored images: fluorescence of chlorophyll, SYTO 9, and labeled Cas9 proteins are shown in blue, green, and red, respectively. The scale bar indicates 1 μm

We further confirmed the intracellular presence of Cas9 proteins in cells by examining using confocal laser scanning microscopy (FV3000, Olympus). Microscopic examination of many electroporated cells revealed four cells containing fluorescently labeled Cas9, of which two are displayed in Fig. 3. In these cells, the fluorescently labeled Cas9 protein (red) seemed to be intracellular. On comparison, microscopic examination of many non-electroporated cells revealed four fluorescently labeled cells, but for those, red fluorescence was detected at the periphery of the cells, indicating that fluorescently labeled Cas9 was not imported in non-electroporated cells.

### *KJFTSY* gene knockout via DNA-free CRISPR

**Fig. 4:**
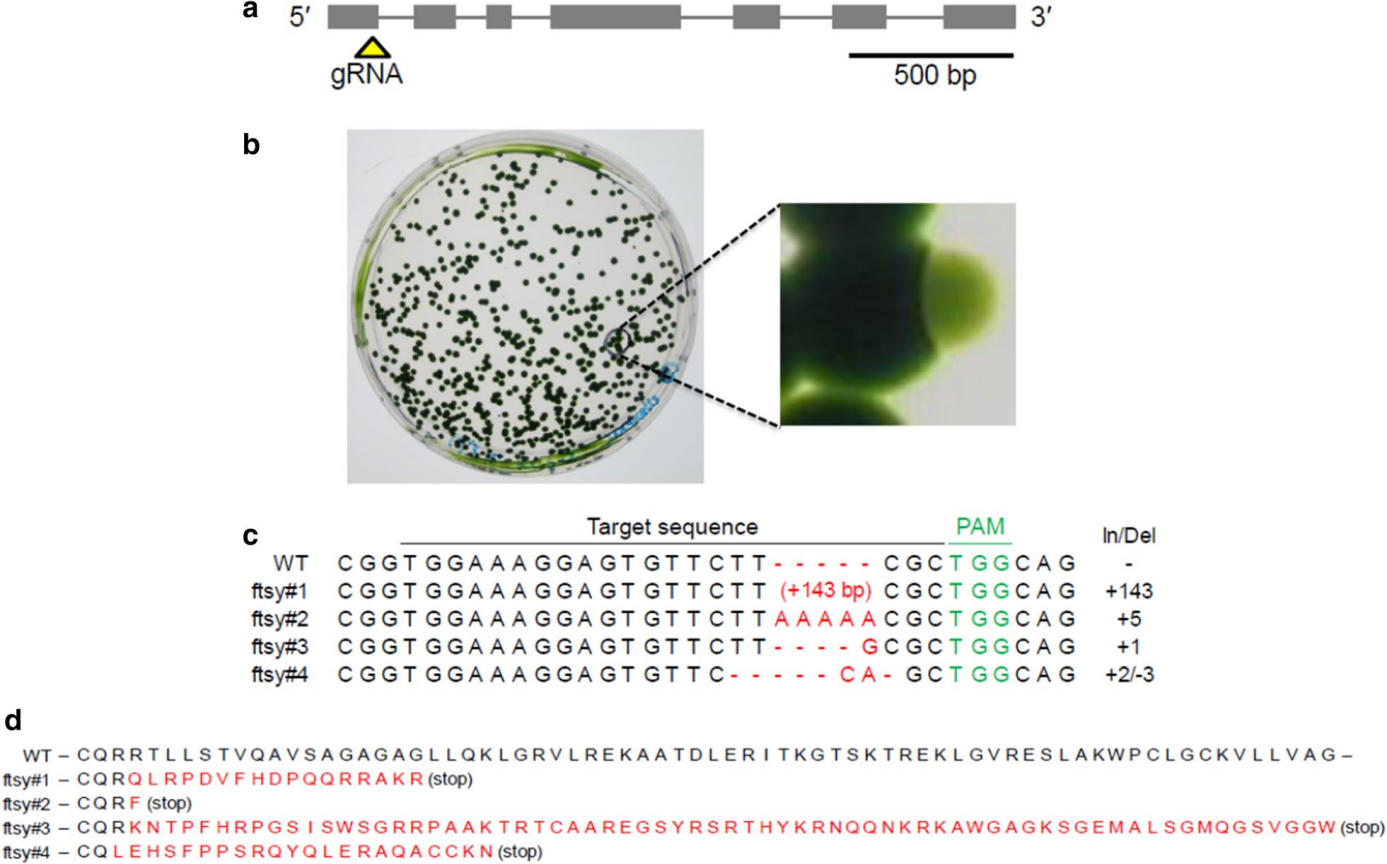
Isolation of *KJFTSY* knockout lines using DNA-free genome editing. **a** Structure of the *KJFTSY* gene and gRNA-targeting site. Gray boxes represent exons; yellow triangle represents the gRNA-targeting site. **b** Color selection of a *KJFTSY* knockout cell. After electroporation of Cas9-gRNA RNP, electroporated cells were spread on a plate to obtain single colonies. An enlarged view of a *KJFTSY* knockout colony is also shown. The photograph was taken after a 7-day incubation in dark followed by a 14-day incubation in light. **c** The gRNA target sequence in the wild-type *KJFTSY* gene and the corresponding sequences in four *FTSY* knockout lines. Red letters indicate inserted nucleotides. The PAM sequence is shown in green. **d** Partial amino acid sequence of wild-type KJFTSY and those of KJFTSY knockdown lines. The red letters indicate amino acid sequences generated by frameshift mutations

To demonstrate successful genome editing in *Coccomyxa* sp. strain KJ, we chose *KJFTSY* as the target gene. FTSY is required for post-translational integration of light-harvesting chlorophyll *a/b*-binding proteins into thylakoid membranes. Thus, the loss of the FTSY function results in a substantial loss of antenna chlorophylls, and mutant colonies exhibit a pale green color [22, 27]. We designed a CRISPR RNA (crRNA) targeting a unique sequence in the 5′ region of *KJFTSY* (Fig. 4a), and chemically synthesized crRNA and trans-activating crRNA (tracrRNA). The purified Cas9 protein was then conjugated with gRNA (tracrRNA plus crRNA), and the RNP complex was delivered into strain KJ cells by electroporation using the “optimum” conditions described above. After the electroporation, the cell suspension was cultured in liquid medium in the dark for 2 or 7 days. Because Xiang et al. [28] reported that the Cas9-gRNA RNP activity increases in a temperature-dependent manner from 30 to 39 °C, the incubation temperature in dark was set to 35 °C, a temperature at which cell proliferation was not severely affected. In the first experiment with a 7-day dark incubation period after electroporation, one pale green colony appeared at a frequency of 0.01% (Fig. 4b, Table 1). In the second experiment, we used four different conditions for Cas9-gRNA RNP-mediated mutagenesis: in the first and second conditions, the poring pulse was at 7,500 V cm^−1^ and the dark incubation period was 2 or 7 days; while in the third and fourth conditions, the poring pulse was at 5000 V cm^−1^ and the dark incubation period was 2 or 7 days. In the second experiment, we isolated one and two knockout lines under the first and second conditions, respectively, but no knockout line was detected under the third and fourth condition (Table 1). The results are in line with the conclusion of the genetic transformation experiments (Additional file 1: Table S1): the electroporation efficiency for strain KJ was higher at 7500 V cm^−1^ than at 5000 V cm^−1^.

**Table 1:** Efficiency of genome editing using Cas9-gRNA RNP

	Expt 1	Expt 2-1	Expt 2-2	Expt 2-3	Expt 2-4
Electric field strength of poring pulse (V cm^−1^)	7500	7500		5000	
Dark incubation period (day)	7	2	7	2	7
Total number of colonies examined	10,760	11,724	11,892	12,306	11,880
Number of knockout lines obtained	1	1	2	0	0
Gene-editing efficiency (%)	0.01	0.01		0	

Thus, a total of four *FTSY* knockout lines were isolated. Sequence analysis revealed that each of the four knockout lines carried four different mutations in the gRNA-targeted sequence, all being frameshift mutations (Fig. 4c, d). All the mutations could have occurred by Cas9-mediated cleavage at three nucleotides upstream of the protospacer adjacent motif (PAM) site. From the observations, we concluded that Cas9-gRNA RNP was successfully applied to edit the genome of *Coccomyxa* sp. strain KJ. Still, the frequency of gene knockout was at a level of 0.01% of electroporated cells (Table 1). This modest frequency may be due to (i) low efficiency in the delivery of Cas9-gRNA RNP into cells (approximately 0.5%; Fig. 2), and (ii) low efficiencies in subsequent steps, namely nuclear translocation of RNP, the double-strand break catalyzed by the RNP, and error-prone repair by non-homologous end joining pathway.

### Phenotype of the *KJFTSY* knockout lines

**Fig. 5:**
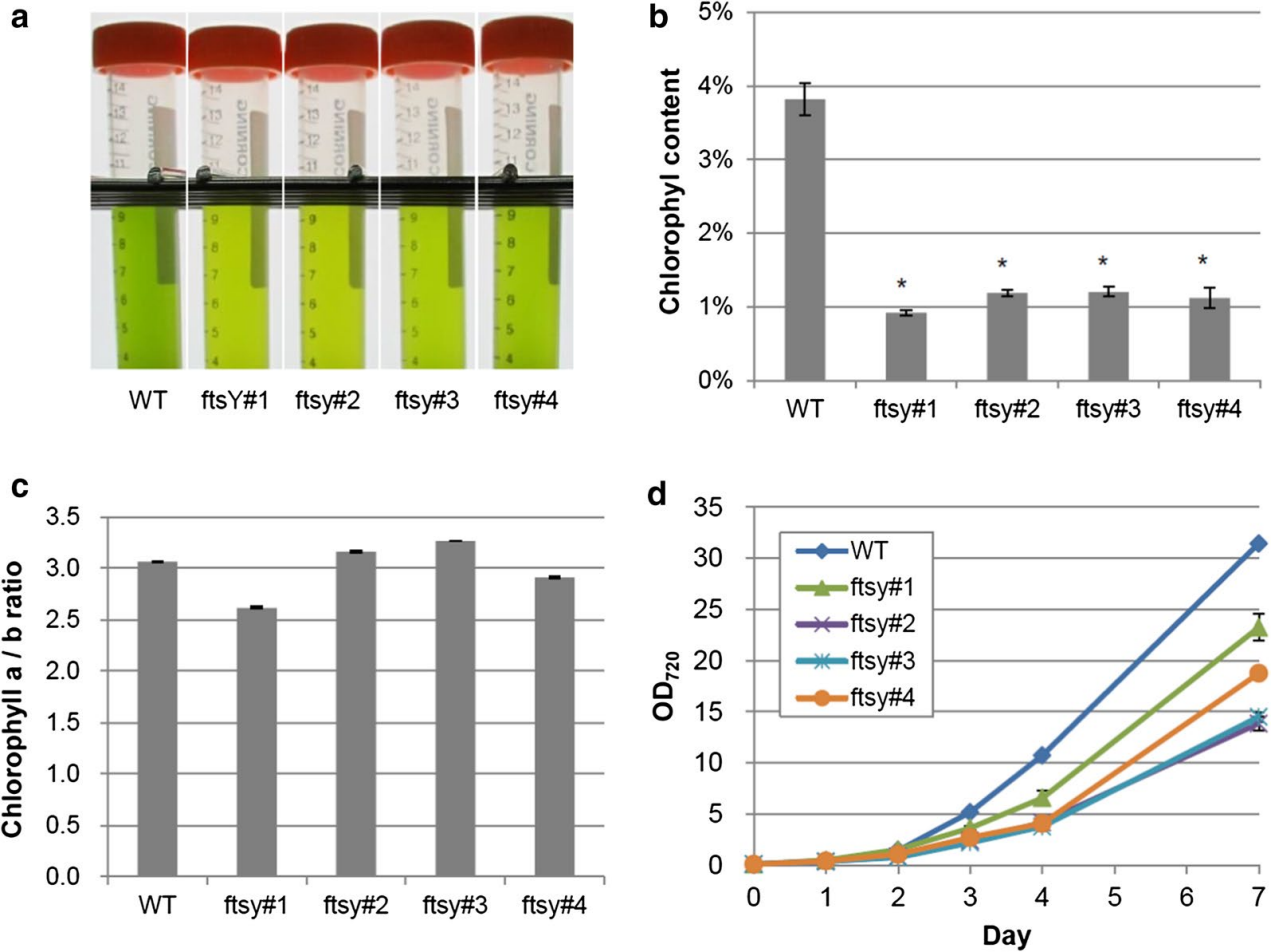
Phenotypic analysis of *KJFTSY* knockout lines. The wild-type strain and four knockout strains were cultured in MA5 liquid medium under continuous light (100 μmol photons m^−2^ s^−1^) with 1% (v/v) CO_2_ bubbling. The photograph of 7-day cultures were taken after adjusting their densities to OD_720_ = 5.0 (**a**). Chlorophyll content per algal dry weight (w/w) (**b**) and *a/b* ratio (**c**) were quantified at day 4 while growth curves (**d**) were followed for 7 days. Each experiment was conducted in biological triplicate, and the mean ± SE were calculated. The asterisks on the top of the histograms indicate a statistically significant difference of *P* < 0.01 using Student’s *t* test

Next, we characterized chlorophyll contents and growth rates of the *KJFTSY* knockout lines. The chlorophyll contents in all knockout lines decreased to < 30% of that in the wild-type (Fig. 5a, b). However, the ratio of chlorophyll *a*/*b* was not markedly different between the knockout lines and wild-type strain (Fig. 5c). This phenotype was similar to that of an *Arabidopsis* mutant defective in FTSY [29], but different from the *a/b* ratios of *C. reinhardtii FTSY* knockout mutants that were higher than the wild-type ratio [22]. The reasons why the *a/b* ratios were different between the *C. reinhardtii* and *Coccomyxa* mutants are currently unclear; however, the difference in light conditions for growth or distinct functions of FTSY in *C. reinhardtii* and *Coccomyxa* may explain such a difference.

Figure 5d shows the growth curves of the wild-type strain and knockout lines under continuous light (100 μmol photons m^−2^ s^−1^). All knockout lines grew at slower rates than the wild-type strain. These results together with those of unchanged chlorophyll *a/b* ratio in the *FTSY* knockout lines suggested that not only the contents of major antenna complexes but also those of minor core antenna and reaction center complexes were diminished in the *FTSY* knockout lines. This possibility should be investigated in further studies.

## Conclusion

To the best of our knowledge, this is the first report of successful application of CRISPR/Cas9 to an industrial green microalga. Using the developed procedure, we have succeeded in isolating *Coccomyxa* mutants with high lipid productivity (manuscript in preparation). However, the application of the procedure is currently limited to readily screenable genes. We are currently working to improve gene knockout frequency to extend the range of applications of CRISPR/Cas9 in *Coccomyxa* and related organisms.

## Methods

### Algal strain and culture conditions

The unicellular green alga *Coccomyxa* sp. strain KJ (Trebouxiophyceae, Chlorophyta) has been deposited in Patent Organism Depositary (IPOD) at National Institute of Technology and Evaluation (NITE) (Chiba, Japan) under deposition number FERM BP-22254.

*Coccomyxa* sp. strain KJ was cultured at 26 °C under continuous light (100 μmol photons m^−2^ s^−1^) in test tubes containing liquid MA5 minimal mineral medium [10] that was bubbled with 2% (v/v) CO_2_. MA5 agar plates were prepared by adding 1.5% (w/v) agar (Bacto Agar, BD Difco, USA) into MA5 medium, and the agar plates were incubated with 1% (v/v) CO_2_ in a growth chamber (LPH-411SPC, NKsystem, Japan).

### Construction of plasmids

The pKE1E plasmid (DDBJ/EMBL/GenBank accession no. LC421406) is a pBluescript SK(-)-based plasmid carrying the promoter and terminator regions of the elongation factor 1-alpha gene from strain KJ (*KJEF1α*). For construction of the pble-PeEGFP-KE1E plasmid (DDBJ/EMBL/GenBank accession no. LC421407), the pKE1E plasmid was digested with NcoI between the promoter and terminator regions, and a DNA fragment, including the *ble* gene, which confers resistance to bleomycin/phleomycin/zeomycin; the linker DNA, encoding the GGSGGR peptide; and the codon-optimized enhanced green fluorescent protein (EGFP), was inserted in the NcoI site using the GeneArt^®^ Seamless Cloning and Assembly Kit (Thermo Fisher Scientific, USA).

### Preparation of Cas9-gRNA RNP

For targeted cleavage of the *FTSY* gene from strain KJ (*KJFTSY*: DDBJ/EMBL/GenBank accession no. LC421405) encoding the chloroplast homolog of signal recognition particle receptor, one crRNA was designed based on the 5′-region sequence of the *KJFTSY* gene (5′-GGAAAGGAGTGTTCTTCGCTGG-3′) followed by PAM (NGG). This sequence was unique compared with the rest of the genome sequence. crRNA and TracrRNA were chemically synthesized by FASMAC (Japan).

Purified recombinant Cas9 protein (3 μg μL^−1^) was purchased from NIPPON GENE (Japan). To prevent arcing during electroporation, 150 μg of the Cas9 protein was diluted tenfold with RNase-free water, the protein was concentrated using an Amicon^®^ ultra centrifugal filter (100 K, Millipore Corporation, USA), and then diluted threefold with RNase-free water to reduce the concentration of NaCl to < 10 mM according to the manufacturer’s instructions. The final concentration of the Cas9 protein was 10 μg μL^−1^. For preparing Cas9-gRNA RNP, 1 µL of Cas9 protein solution, 1 μL of tracrRNA (200 pmol μL^−1^), and 1 μL of crRNA (200 pmol μL^−1^) were added to 3 μL of RNase-free water, according to the method used by Baek et al. [22]. The resulting mixture (total 6 μL) was incubated at 26 °C for > 10 min before electroporation.

### DNA or protein delivery into strain KJ cells

For electroporation, strain KJ cells, precultured in MA5 medium supplemented with 0.6 M sorbitol under 12-h/12-h light/dark conditions for 5 days, were transferred into 50 mL of fresh medium of the same composition at an initial OD_720_ of 0.005. The cells were then cultured for an additional 6–7 days. Cells in 50 mL of the cultures were then harvested by centrifugation (3000 rpm at 25 °C for 5 min), washed twice in 5 mM MES buffer (pH 5.5), and suspended in 200 μL of MAX Efficiency™ Transformation Reagent for Algae (A24229, Thermo Fisher Scientific, USA) to cell densities between 1.2 and 4.0 × 10^9^ cells mL^−1^. The cell suspensions were subsequently diluted with MAX Efficiency™ Transformation Reagent for Algae at a final density of 1.0 × 10^9^ cells mL^−1^ and were kept on ice prior to use for electroporation.

For delivery of the pble-PeEGFP-KE1E plasmid DNA in strain KJ cells, a 30-μL aliquot of the suspension containing 3.0 × 10^7^ cells, 4 μL of 10% (w/v) glucose, and 1, 3 or 4 μg of the pble-PeEGFP-KE1E plasmid DNA, were mixed in a chilled 2-mm-gap electroporation cuvette (EC-002S, Nepa Gene Co., Ltd, Japan) and incubated at 16 °C for 2 min. Electroporation was performed with a square electric pulse system (ELEPO21, Nepa Gene Co., Ltd, Japan) using a three-step pulse protocol, as described below.

The first step of the protocol included 1–4 consecutive 50-ms-interval application(s) of the “poring” pulse(s) with an electric field strength between 3000 and 11,500 V cm^−1^ and a pulse width between 2.5 and 15 ms; the second step included four consecutive 50-ms-interval applications of the “transfer” pulses with an electric field strength between 5 and 500 V cm^−1^ and a pulse width between 1.0 and 99.9 ms; and the third step included four consecutive 50-ms-interval applications of the “reverse transfer” pulses with an electric field strength between − 5 and − 500 V cm^−1^ and a pulse width between 1.0 and 99.9 ms.

For delivery of Cas9-gRNA RNP to strain KJ cells, a 30-μL aliquot of the suspension containing 3.0 × 10^7^ cells, 4 μL of 10% (w/v) glucose, and Cas9-gRNA RNP (containing 10 μg of Cas9), was added to a chilled 2-mm-gap electroporation cuvette, and electroporation was performed as described above, except that a single poring pulse with an electric field strength of 7500 V cm^−1^ and a pulse width of 2.5 ms was applied, followed by four consecutive transfer pulses (electric field strength: 250 V cm^−1^; pulse width: 50 ms) and four consecutive reverse transfer pulses (electric field strength: − 250 V cm^−1^; pulse width: 50 ms). The intervals between the pulses were 50 ms.

Immediately after electroporation with DNA or RNP, 2-mL MA5 medium containing 1% (w/v) glucose was added in the cuvette, the cell suspension was transferred into a 12-mL tube (T406-2A, BM Equipment Co., Ltd, Japan), and incubated with shaking (100–150 strokes min^−1^) at 35 °C under dark conditions. When DNA of the pble-PeEGFP-KE1E plasmid was introduced with electroporation, the cell suspension was incubated for 1 day, and the total amount of the suspension was spread on a MA5 plate containing 200 µg mL^−1^ Zeocin™ (Thermo Fisher Scientific, USA). When Cas9-gRNA RNP was introduced with electroporation, the cell suspension incubated for 2 or 7 days under dark conditions was diluted and spread on MA5 agar plates at a density of 1000–2000 cells per plate to obtain single colonies for color selection. If necessary, the single colonies were transferred to gridded master plates for further investigation.

### Fluorescent analysis using fluorescently labeled Cas9 protein

To detect intracellular localization of the Cas9 protein after electroporation, purified Cas9 protein was labeled using the HiLyte Fluor™ 555 labeling kit (Dojindo Molecular Technologies, USA), according to the manufacturer’s instructions. Approximately, 6 μg of fluorescently labeled Cas9 protein were added to 3.0 × 10^7^ strain KJ cells in a chilled 2-mm-gap cuvette, and the cells were electroporated using the above-mentioned conditions for electroporation of Cas9-gRNA RNP. As a control, cells and fluorescently labeled Cas9 protein were mixed in an electroporation cuvette, but no electric pulse was applied. The electroporated and non-electroporated cells in the electroporation cuvette were incubated for few hours at room temperature and washed 3 or 5 times thoroughly with PBS buffer to eliminate the fluorescently labeled Cas9 protein adsorbed on cell surfaces. FACS analysis was then performed using a MoFlo^®^ Astrios™ flow cytometer (Beckman Coulter, USA). Signals from the fluorescently labeled Cas9 protein were detected using a 561-nm laser light and a 614/20-nm band-pass filter [center wavelength at 614 nm and full width at half maximum of 20 nm].

Before microscopic observation, electroporated and non-electroporated cells mixed with the fluorescently labeled Cas9 protein were stained with SYTO 9 (Thermo Fisher Scientific, USA) for visualization of the nucleus. Fluorescent imaging was performed using a confocal laser scanning microscope (FV3000, Olympus, Japan) with a 100 × objective lens to determine localization of the Cas9 protein in strain KJ cells. Fluorescence of SYTO 9, fluorescently labeled Cas9 protein, and chlorophyll were detected using laser excitation of 488 nm combined with a 520/40 nm band-pass filter, 561 nm combined with a 595/50 band-pass filter, and 640 nm combined with a 710/100 band-pass filter, respectively. Images were acquired using DP80 (Olympus, Japan).

### DNA sequencing of gene knockout lines

For extraction of genomic DNA, cells derived from a single colony were transferred to TE buffer [10 mM Tris–HCl (pH 8.0) and 1 mM EDTA] in a PCR tube and frozen in a deep freezer at − 80 °C for at least one night. After thawing the cells at room temperature, cell debris was precipitated by centrifugation, and the supernatants were used as a template for PCR amplification. Genomic PCR was performed with GoTaq Master Mix (Promega, USA) and specific primers, FY_F1 (5′-AAGCAAGCACTAGCGCAGAC-3′) and FY_R1 (5′-TCCCACTGAAAGTGGTGGAC-3′), which were designed based on the genomic sequence flanking the target site within the *KJFTSY* gene. Amplification conditions were as follows: initial denaturation at 95 °C for 1 min, 33 cycles of 30 s at 95 °C and 25 s at 60 °C, and final extension at 68 °C for 3 min. Nucleotide sequences were determined by FASMAC (Japan).

### Measurement of chlorophyll content

For quantitative estimation of the chlorophyll content, strain KJ cells and several knockout lines of strain KJ were grown in MA5 medium and harvested by centrifugation. The supernatants were removed, and the precipitated cells were resuspended in 1 mL of *N*,*N*-dimethylformamide and heated at 50 °C for 5 min to extract pigments. Cell debris was precipitated by centrifugation, and chlorophyll contents in the supernatants were measured. Chlorophyll absorbance was measured at 663.8, 646.8, and 750 nm using a spectrophotometer (U-4100, HITACHI, Japan). The chlorophyll content was calculated according to Porra’s formula [30].

## Additional file

**Additional file 1: Figure S1.** Effect of intensities of poring pulse energy on cell viability of strain KJ. **Table S1.** Effect of electroporation conditions on genetic transformation efficiency of strain KJ.

## Abbreviations

CRISPR: clustered regularly interspaced short palindromic repeats
gRNA: guide RNA
RNP: ribonucleoprotein
EGFP: enhanced green fluorescent protein
crRNA: CRISPR RNA
tracrRNA: trans-activating crRNA
PAM: protospacer adjacent motif
FACS: fluorescence-activated cell sorting
