# Supplementary material for: Cas9-guide RNA ribonucleoprotein-induced genome editing in the industrial green alga Coccomyxa sp. strain KJ
Source: Biotechnol Biofuels. 2018 Dec 10;11:326. doi: 10.1186/s13068-018-1327-1 (PMC6287348; doi:10.1186/s13068-018-1327-1)
Supplement: Supplementary file 1 — Additional file 1: Figure S1. Effect of intensities of poring pulse energy on cell viability of strain KJ. Table S1. Effect of electroporation conditions on genetic transformation efficiency of strain KJ. [file 13068_2018_1327_MOESM1_ESM.docx]

**Additional file**

Cas9-guide RNA ribonucleoprotein-induced genome editing in the industrial green alga *Coccomyxa* sp. strain KJ

Yuya Yoshimitsu ^1^, Jun Abe ^2^, Shigeaki Harayama ^2, 3*^

**Table S1.** Effect of electroporation conditions on genetic transformation efficiency of strain KJ

Table S1 (continued)


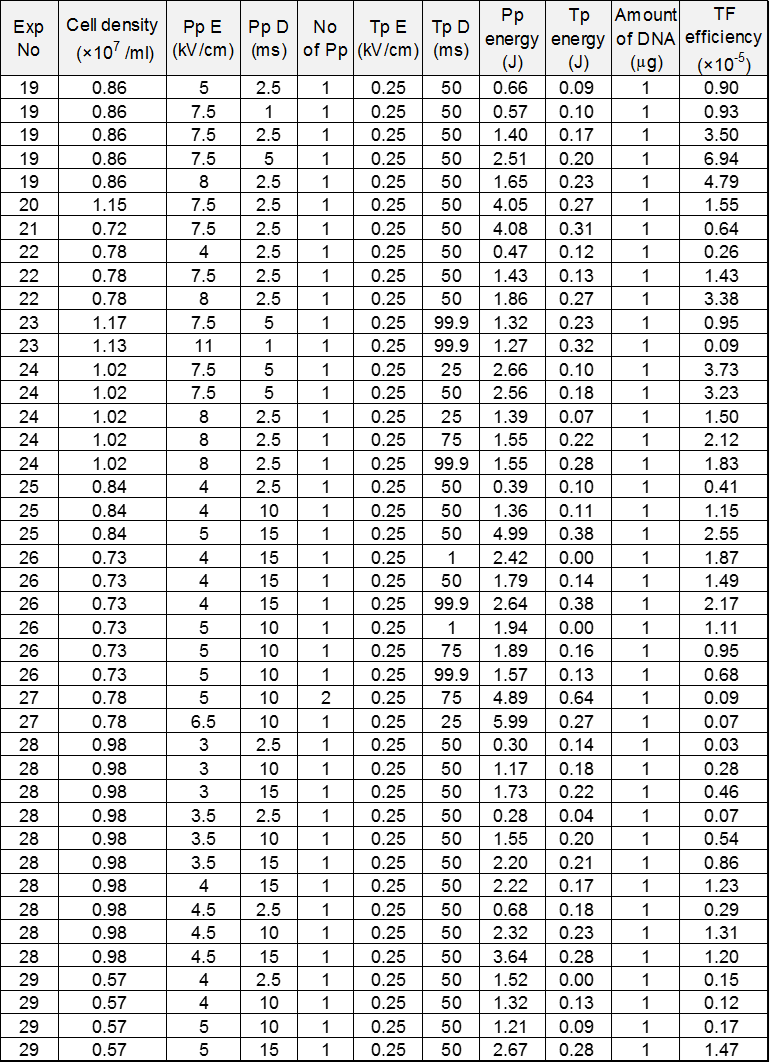


**Exp No**: Experiment number. Data with the same experiment number were obtained using cells from the same culture. **Cell density**: cell density at harvest. **Pp E**: Field strength of poring pulse(s). **Pp D**: duration of poring pulse(s). **No of Pp**: number of poring pulses. **Tp E**: Field strength of transfer pulses consisting of 4× “forward” and 4× “reverse” transfer pulses. **Tp D**: duration of each transfer pulse. **Pp energy**: energy (Joule) delivered by poring pulse(s). **Tp energy**: energy (Joule) delivered by transfer pulses. **Amount of DNA**: amount of DNA used per electroporation. **TF efficiency**: transformation efficiency was calculated by dividing the number of Zeocin^TM^-resistant clones by the number of input cells (3.0 × 10^7^). Pp energy and Tp energy in Joule were calculated using the equation: (volt per 0.2 cm)× (ampere) × (scare pulse width in second) × (number of pulses).


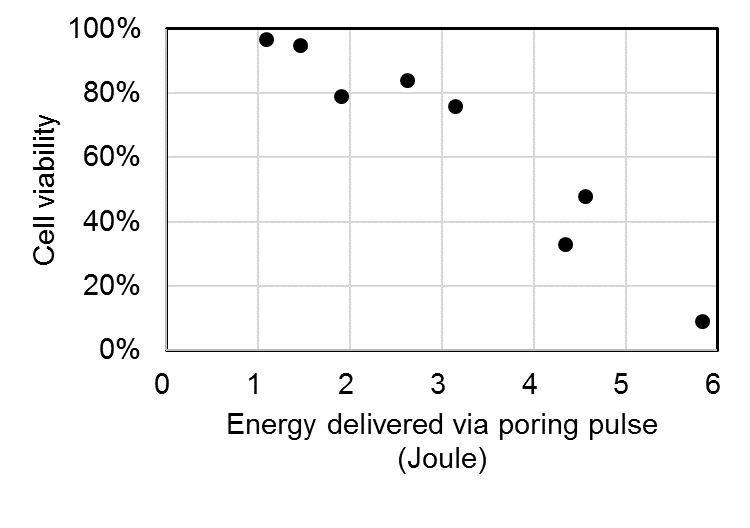


**Figure S1** Effect of intensities of poring pulse energy on cell viability of strain KJ

Electroporation was conducted under the following conditions: for poring pulses, electric field strength was either at 6,500 or 7,500 V cm^−1^, pulse width was between 2.5 and 10 ms, and a single pulse was applied; for transfer pulses, the following single conditions were used: four consecutive “forward” and four consecutive “reverse” pulses with 50 msec pulse width and 50 msec pulse intervals were applied at electric field strength of 250 V cm^−1^. Energies delivered via poring pulse were recorded for each electroporation. After electroporation of 3.0 × 10^7^ cells with 1 µg DNA of the pble-PeEGFP-KE1E plasmid, the cell suspension was spread on MA5 agar plates at a density of 500–1,000 cells/plate; the means of colony numbers appeared on three plates was calculated and the cell viability was evaluated by dividing the colony number of electroporated cells by the colony number of non-electroporated cells.
